# Supplementary material for: Signalling mechanisms and agricultural applications of (Z)-3-hexenyl butyrate-mediated stomatal closure
Source: Hortic Res. 2023 Nov 28;11(1):uhad248. doi: 10.1093/hr/uhad248 (PMC10794947; doi:10.1093/hr/uhad248)
Supplement: Web_Material_uhad248 [file web_material_uhad248.zip › Supporting Information_reviewed 2_clean.pptx]

## Slide 1
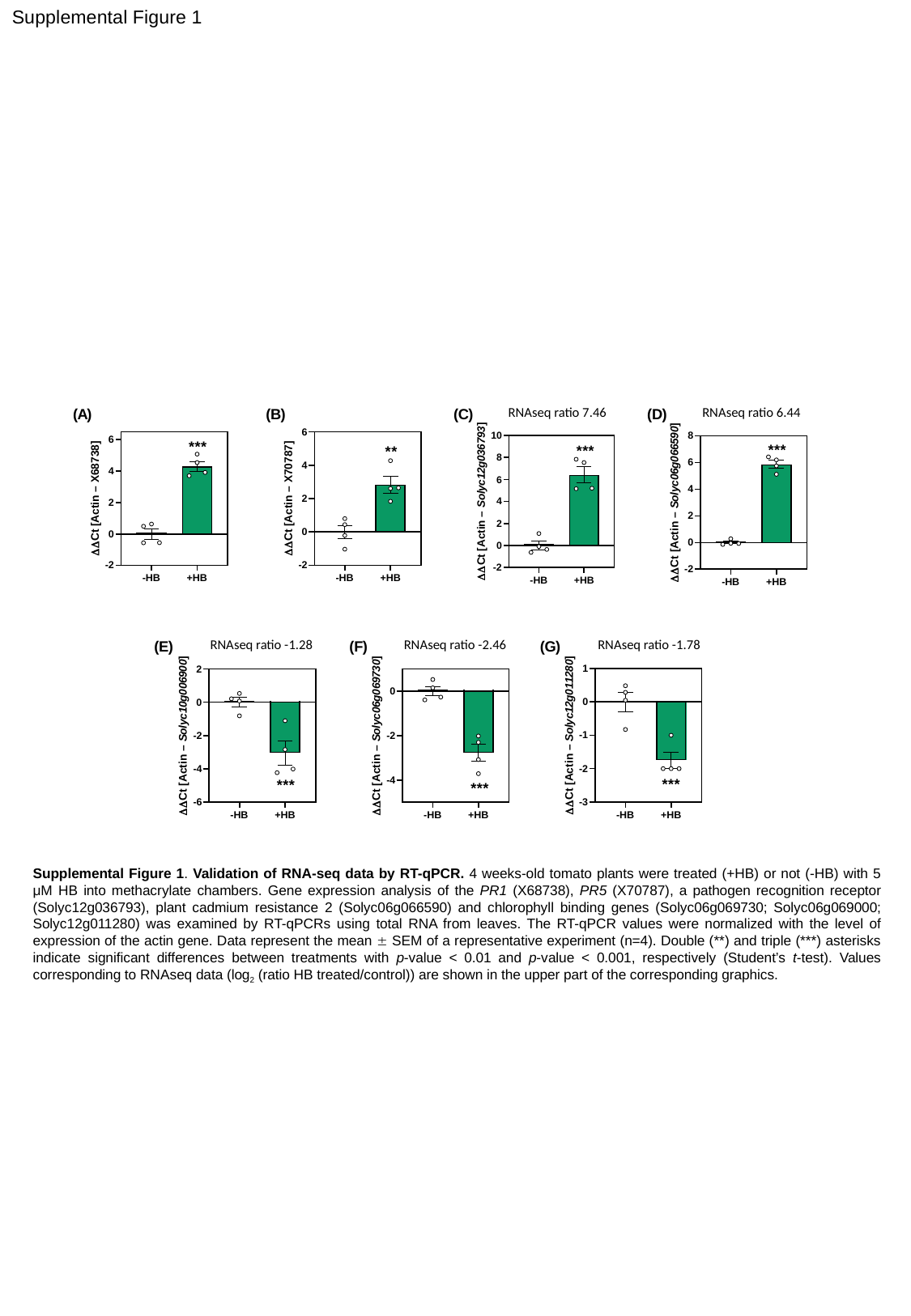

Supplemental Figure 1
RNAseq ratio 7.46
RNAseq ratio 6.44
RNAseq ratio -1.28
RNAseq ratio -1.78
RNAseq ratio -2.46
Supplemental Figure 1. Validation of RNA-seq data by RT-qPCR. 4 weeks-old tomato plants were treated (+HB) or not (-HB) with 5 μM HB into methacrylate chambers. Gene expression analysis of the PR1 (X68738), PR5 (X70787), a pathogen recognition receptor (Solyc12g036793), plant cadmium resistance 2 (Solyc06g066590) and chlorophyll binding genes (Solyc06g069730; Solyc06g069000; Solyc12g011280) was examined by RT-qPCRs using total RNA from leaves. The RT-qPCR values were normalized with the level of expression of the actin gene. Data represent the mean ± SEM of a representative experiment (n=4). Double (**) and triple (***) asterisks indicate significant differences between treatments with p-value < 0.01 and p-value < 0.001, respectively (Student’s t-test). Values corresponding to RNAseq data (log2 (ratio HB treated/control)) are shown in the upper part of the corresponding graphics.

## Slide 2
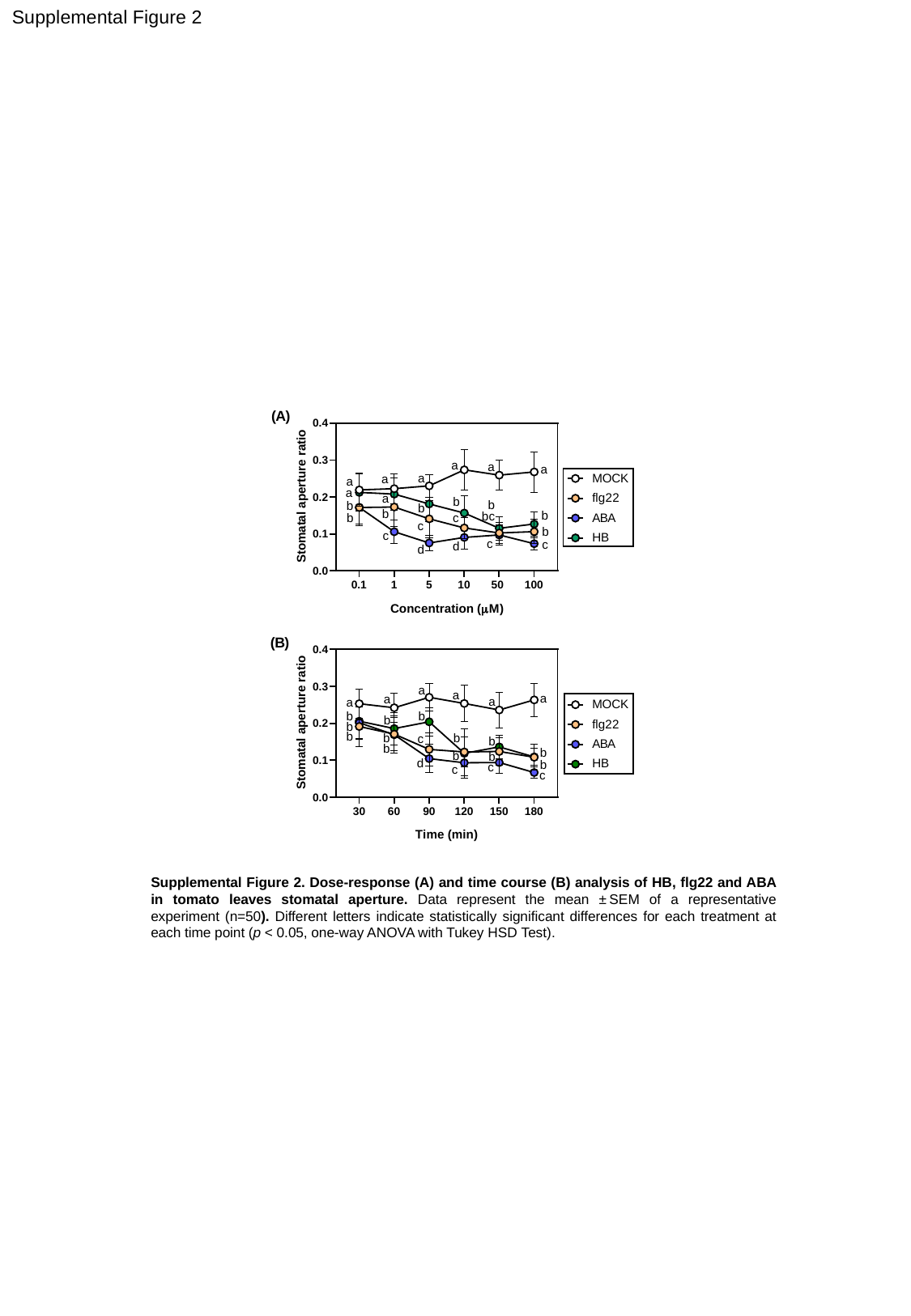

Supplemental Figure 2
Supplemental Figure 2. Dose-response (A) and time course (B) analysis of HB, flg22 and ABA in tomato leaves stomatal aperture. Data represent the mean ± SEM of a representative experiment (n=50). Different letters indicate statistically significant differences for each treatment at each time point (p < 0.05, one-way ANOVA with Tukey HSD Test).

## Slide 3
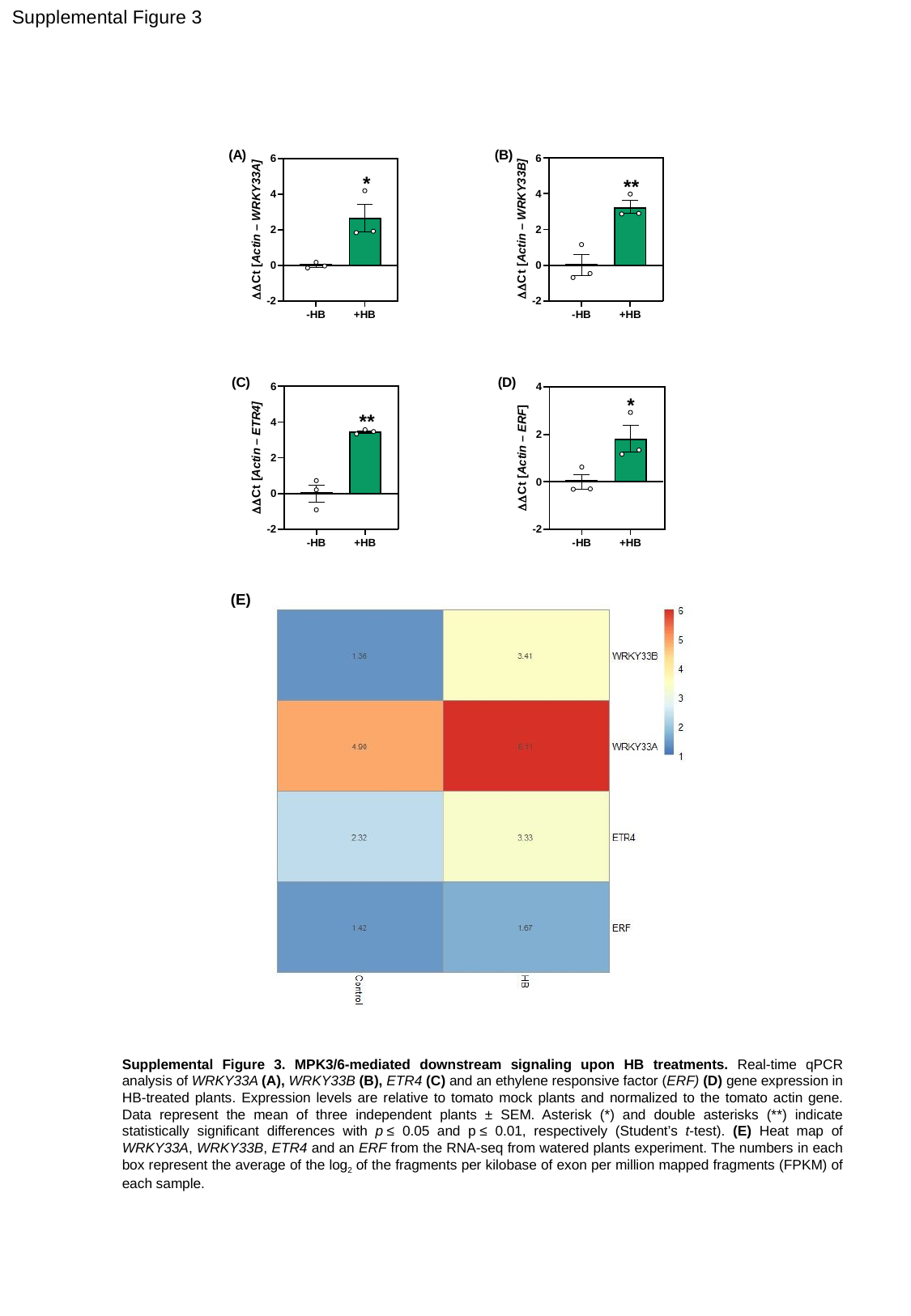

Supplemental Figure 3
(E)
Supplemental Figure 3. MPK3/6-mediated downstream signaling upon HB treatments. Real-time qPCR analysis of WRKY33A (A), WRKY33B (B), ETR4 (C) and an ethylene responsive factor (ERF) (D) gene expression in HB-treated plants. Expression levels are relative to tomato mock plants and normalized to the tomato actin gene. Data represent the mean of three independent plants ± SEM. Asterisk (*) and double asterisks (**) indicate statistically significant differences with p ≤ 0.05 and p ≤ 0.01, respectively (Student’s t-test). (E) Heat map of WRKY33A, WRKY33B, ETR4 and an ERF from the RNA-seq from watered plants experiment. The numbers in each box represent the average of the log2 of the fragments per kilobase of exon per million mapped fragments (FPKM) of each sample.

## Slide 4
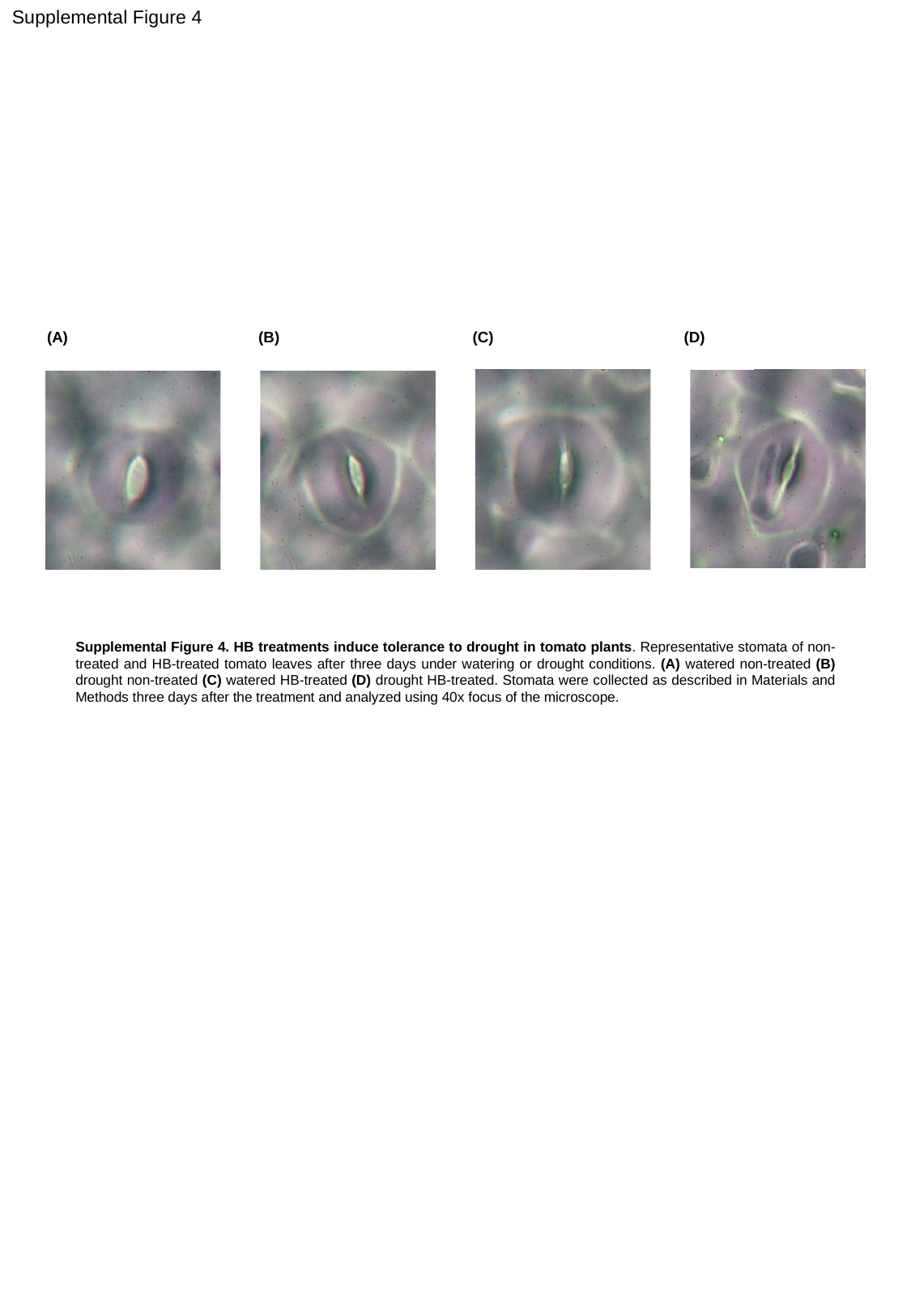

Supplemental Figure 4
(A)
(B)
(C)
(D)
Supplemental Figure 4. HB treatments induce tolerance to drought in tomato plants. Representative stomata of non-treated and HB-treated tomato leaves after three days under watering or drought conditions. (A) watered non-treated (B) drought non-treated (C) watered HB-treated (D) drought HB-treated. Stomata were collected as described in Materials and Methods three days after the treatment and analyzed using 40x focus of the microscope.

## Slide 5
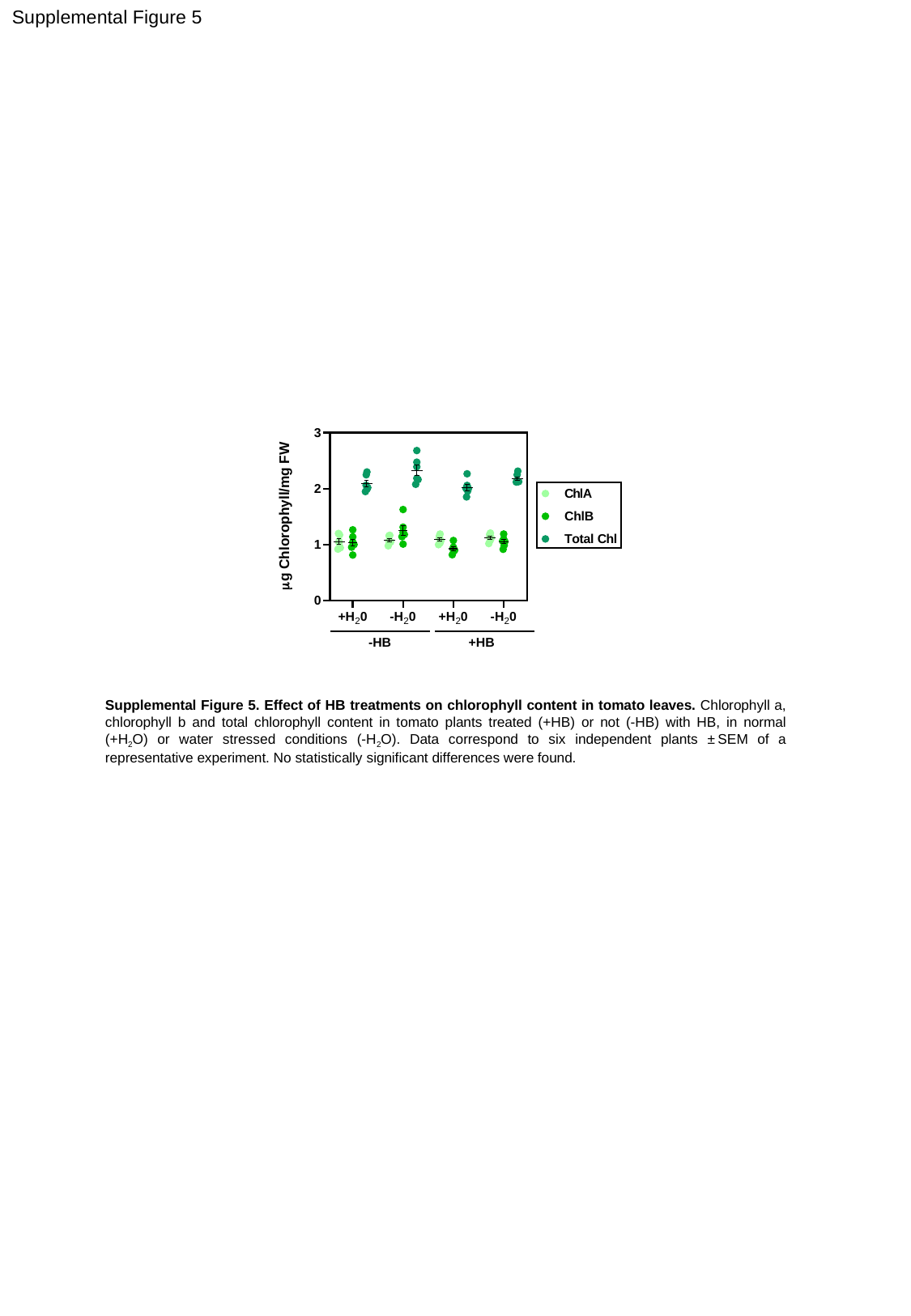

Supplemental Figure 5
Supplemental Figure 5. Effect of HB treatments on chlorophyll content in tomato leaves. Chlorophyll a, chlorophyll b and total chlorophyll content in tomato plants treated (+HB) or not (-HB) with HB, in normal (+H2O) or water stressed conditions (-H2O). Data correspond to six independent plants ± SEM of a representative experiment. No statistically significant differences were found.

## Slide 6
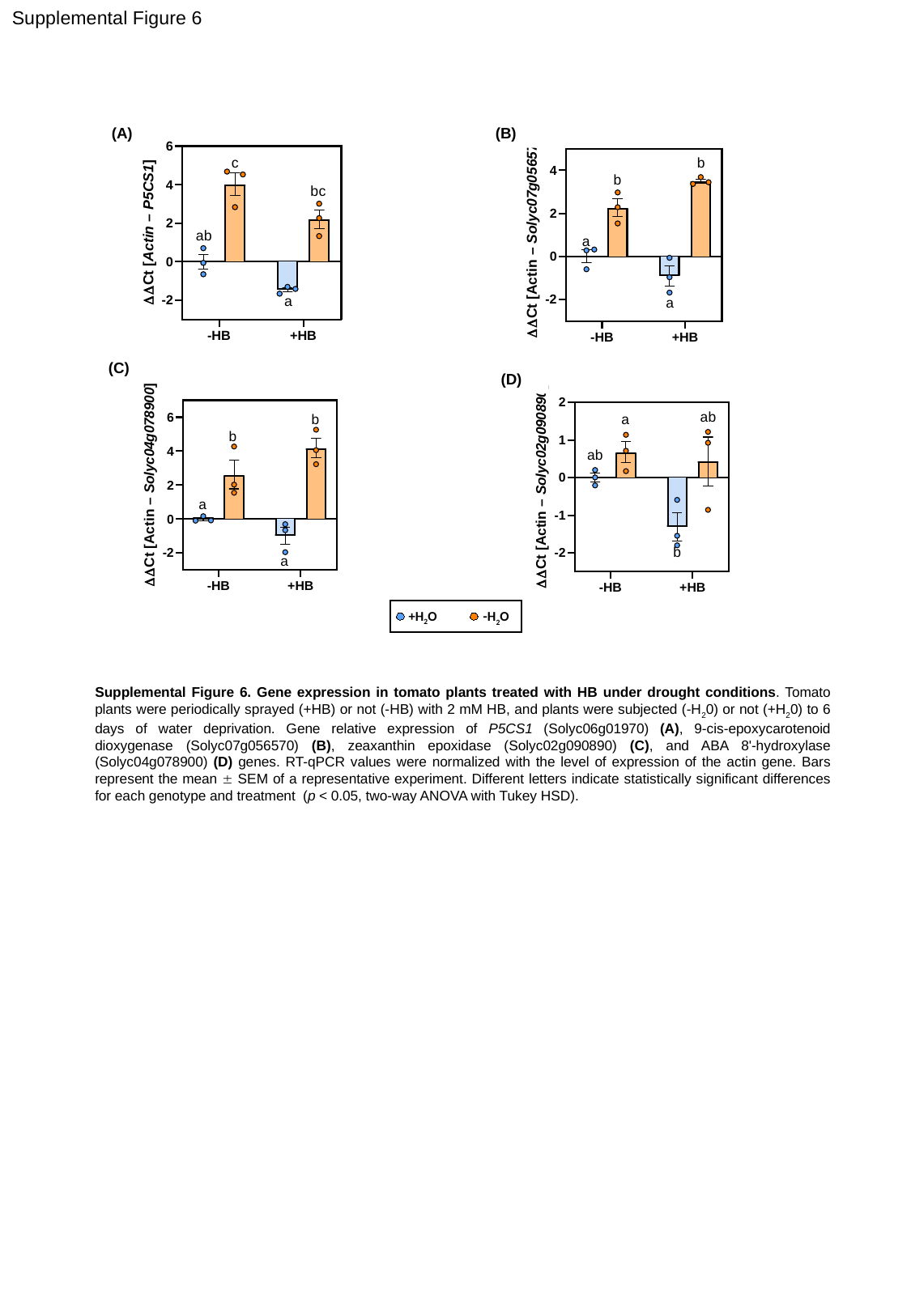

Supplemental Figure 6
(A)
(B)
(C)
(D)
+H2O
-H2O
Supplemental Figure 6. Gene expression in tomato plants treated with HB under drought conditions. Tomato plants were periodically sprayed (+HB) or not (-HB) with 2 mM HB, and plants were subjected (-H20) or not (+H20) to 6 days of water deprivation. Gene relative expression of P5CS1 (Solyc06g01970) (A), 9-cis-epoxycarotenoid dioxygenase (Solyc07g056570) (B), zeaxanthin epoxidase (Solyc02g090890) (C), and ABA 8'-hydroxylase (Solyc04g078900) (D) genes. RT-qPCR values were normalized with the level of expression of the actin gene. Bars represent the mean ± SEM of a representative experiment. Different letters indicate statistically significant differences for each genotype and treatment  (p < 0.05, two-way ANOVA with Tukey HSD).

## Slide 7
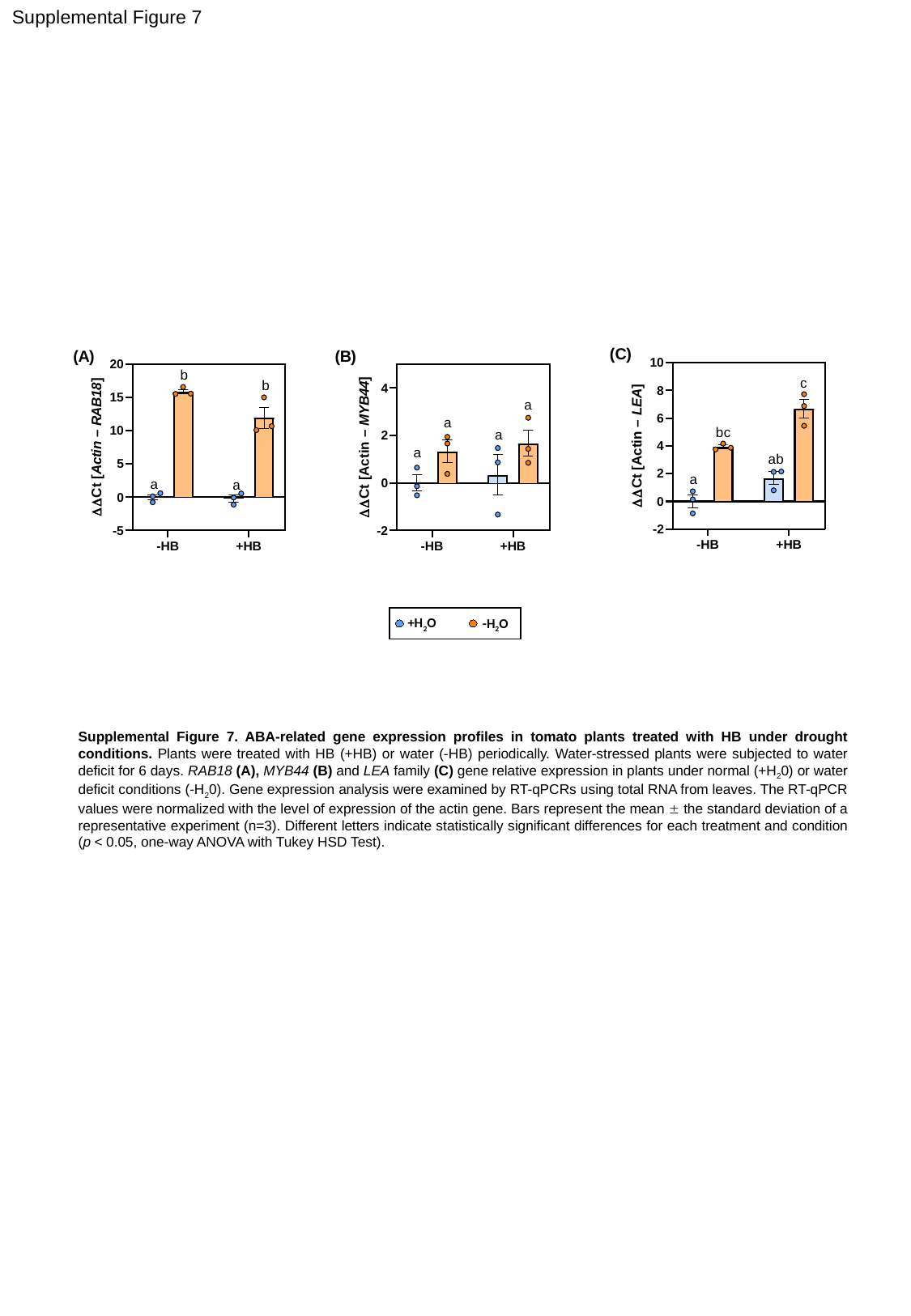

Supplemental Figure 7
+H2O
-H2O
Supplemental Figure 7. ABA-related gene expression profiles in tomato plants treated with HB under drought conditions. Plants were treated with HB (+HB) or water (-HB) periodically. Water-stressed plants were subjected to water deficit for 6 days. RAB18 (A), MYB44 (B) and LEA family (C) gene relative expression in plants under normal (+H20) or water deficit conditions (-H20). Gene expression analysis were examined by RT-qPCRs using total RNA from leaves. The RT-qPCR values were normalized with the level of expression of the actin gene. Bars represent the mean ± the standard deviation of a representative experiment (n=3). Different letters indicate statistically significant differences for each treatment and condition (p < 0.05, one-way ANOVA with Tukey HSD Test).

## Slide 8
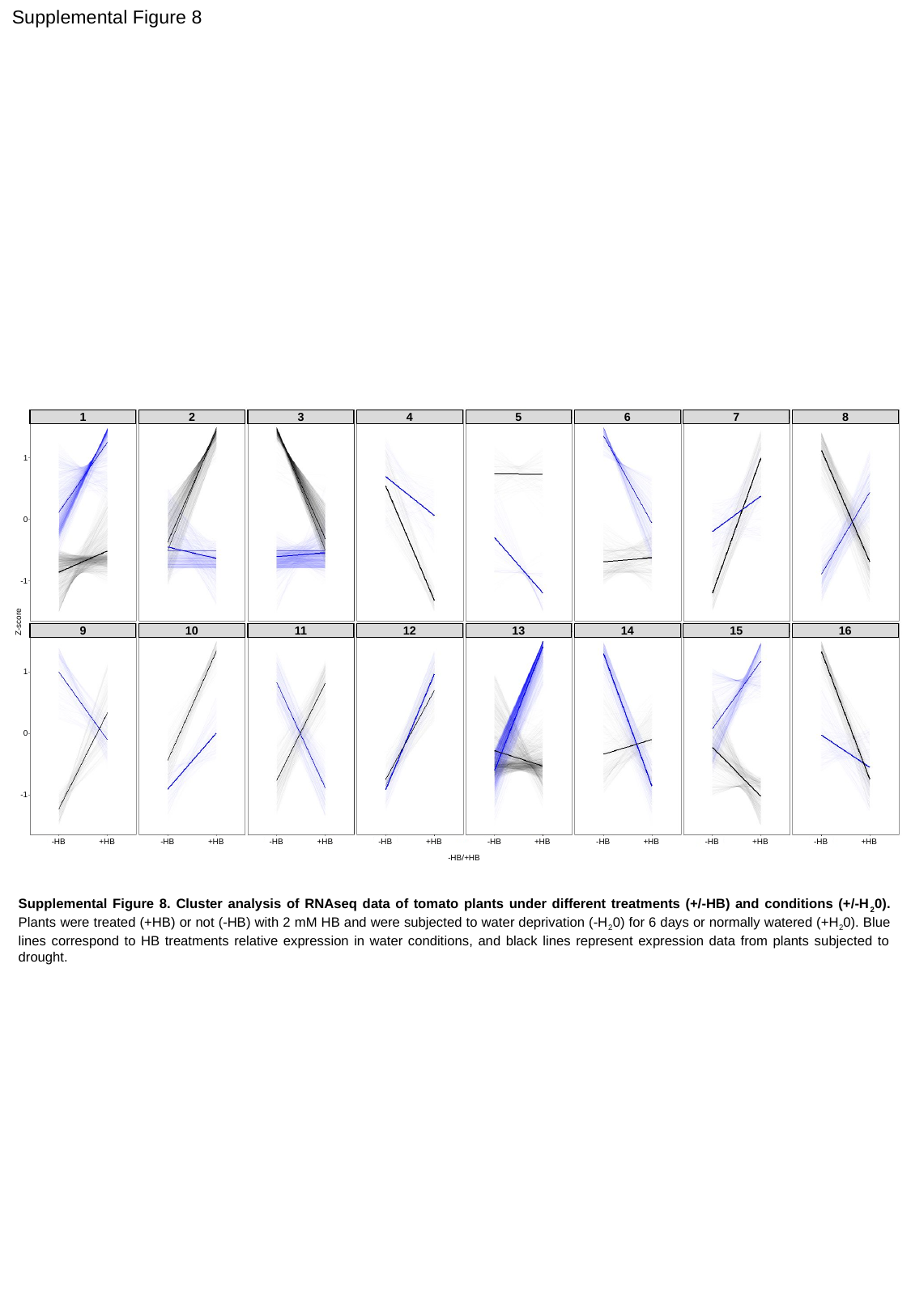

Supplemental Figure 8
1
2
3
4
5
6
7
8
1
0
-1
Z-score
9
10
11
12
13
14
15
16
1
0
-1
-HB
+HB
-HB
+HB
-HB
+HB
-HB
+HB
-HB
+HB
-HB
+HB
-HB
+HB
-HB
+HB
-HB/+HB
Supplemental Figure 8. Cluster analysis of RNAseq data of tomato plants under different treatments (+/-HB) and conditions (+/-H20). Plants were treated (+HB) or not (-HB) with 2 mM HB and were subjected to water deprivation (-H20) for 6 days or normally watered (+H20). Blue lines correspond to HB treatments relative expression in water conditions, and black lines represent expression data from plants subjected to drought.

## Slide 9
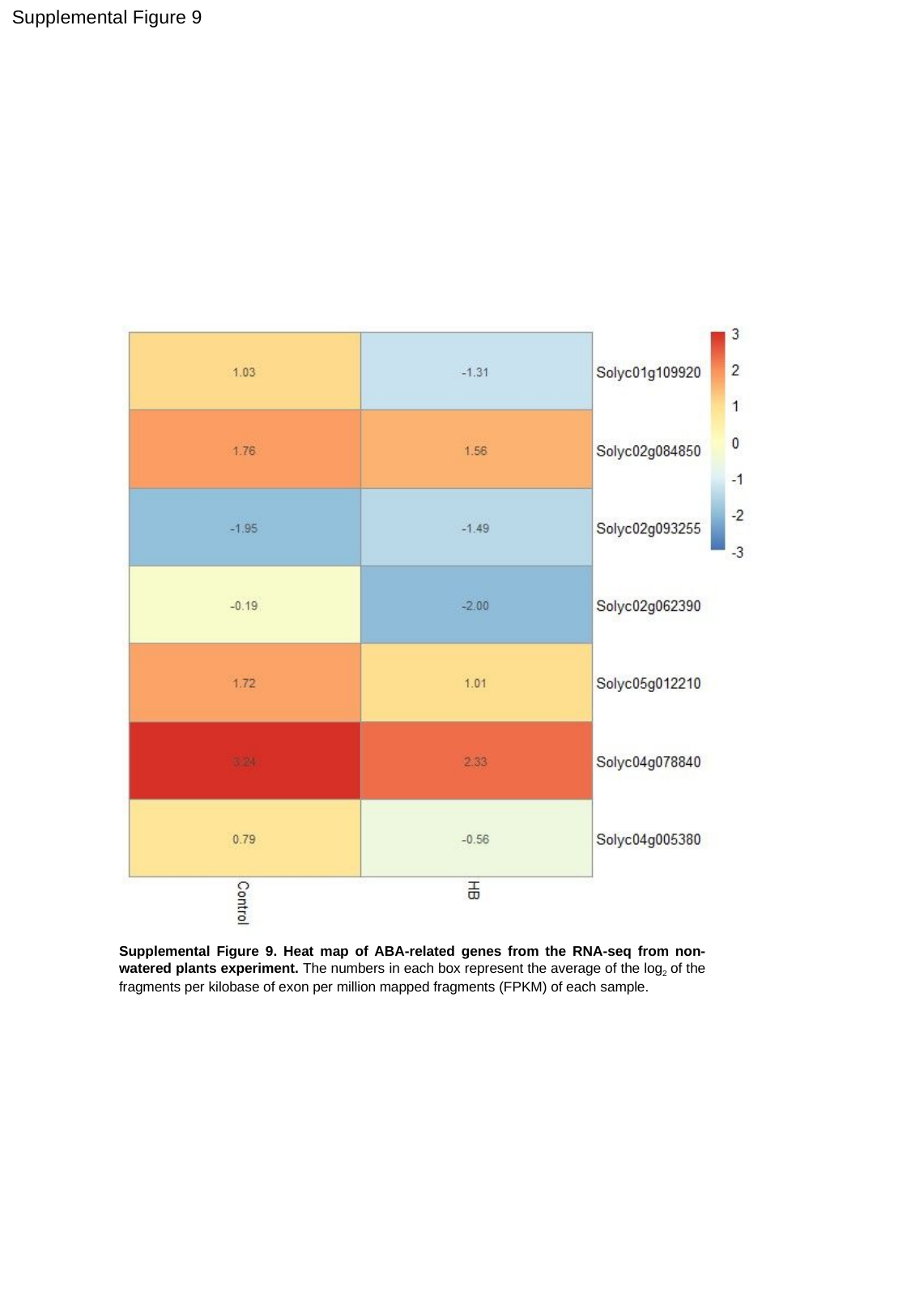

Supplemental Figure 9
Supplemental Figure 9. Heat map of ABA-related genes from the RNA-seq from non-watered plants experiment. The numbers in each box represent the average of the log2 of the fragments per kilobase of exon per million mapped fragments (FPKM) of each sample.

## Slide 10
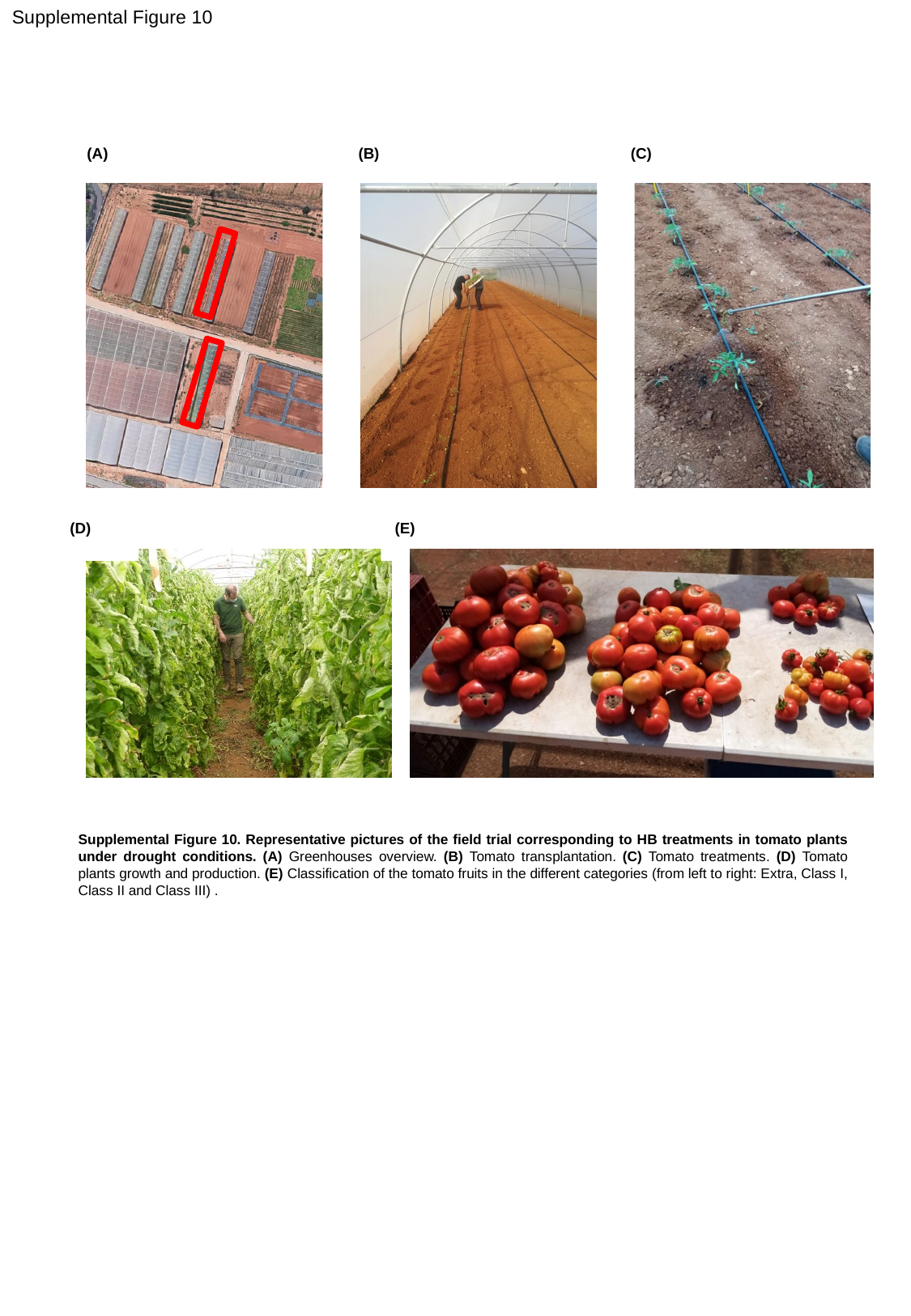

Supplemental Figure 10
(A)
(B)
(C)
(D)
(E)
Supplemental Figure 10. Representative pictures of the field trial corresponding to HB treatments in tomato plants under drought conditions. (A) Greenhouses overview. (B) Tomato transplantation. (C) Tomato treatments. (D) Tomato plants growth and production. (E) Classification of the tomato fruits in the different categories (from left to right: Extra, Class I, Class II and Class III) .

## Slide 11
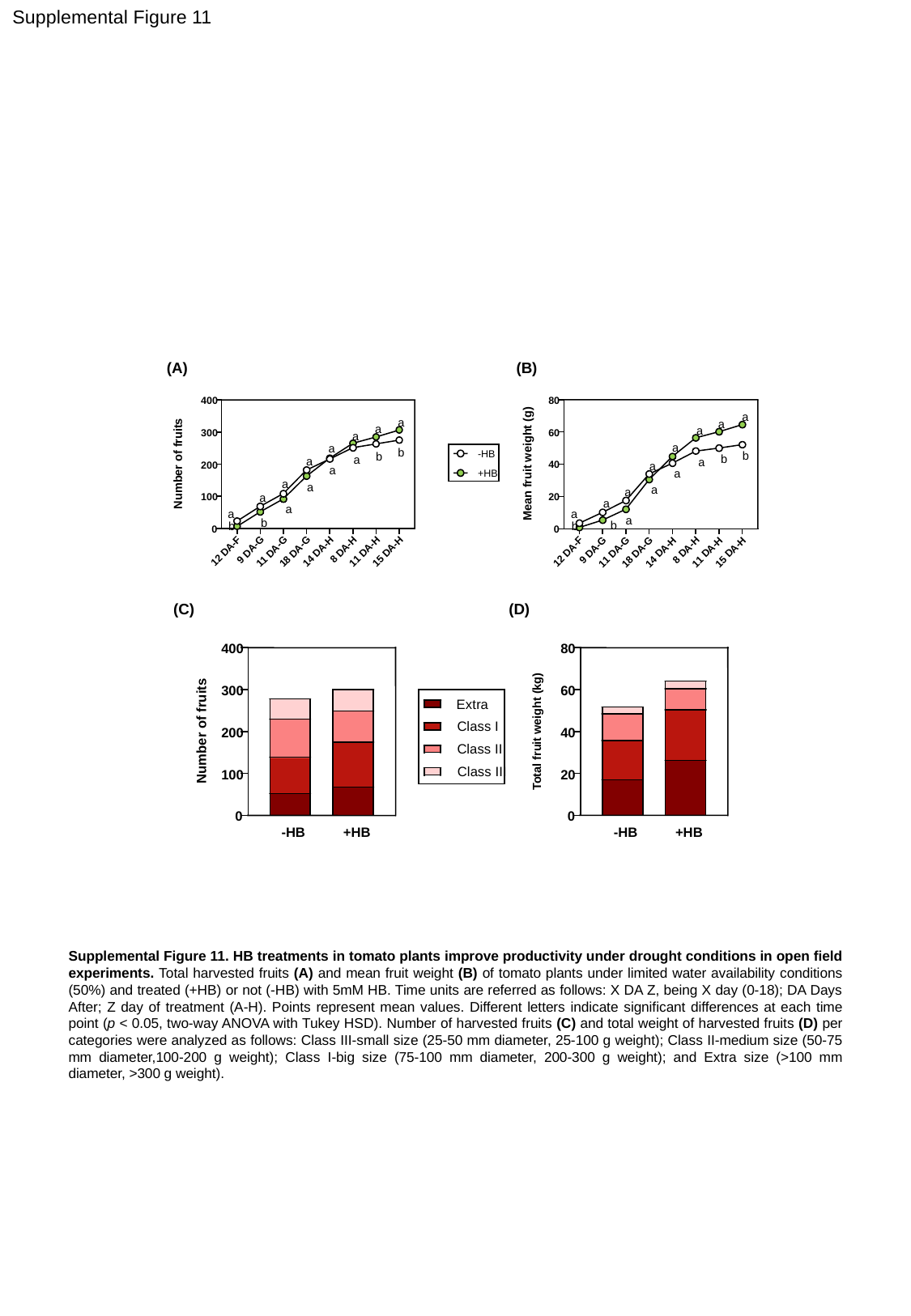

Supplemental Figure 11
(A)
(B)
80
a
a
a
60
a
b
b
a
40
a
a
a
a
20
a
a
a
b
b
0
F
H
H
H
H
G
G
G
-
-
-
-
-
-
-
-
A
A
A
A
A
A
A
A
D
D
D
D
D
D
D
D
2
4
8
1
5
9
1
8
1
1
1
1
1
1
400
a
a
300
a
a
b
b
a
a
200
a
a
a
a
100
a
a
b
b
0
F
H
G
G
G
H
H
H
-
-
-
-
-
-
-
-
A
A
A
A
A
A
A
A
D
D
D
D
D
D
D
D
2
4
8
1
5
9
1
8
1
1
1
1
1
1
-HB
+HB
Number of fruits
Mean fruit weight (g)
(C)
(D)
400
80
60
40
20
0
-HB
+HB
300
Extra
Class I
Number of fruits
200
Total fruit weight (kg)
Class II
Class III
100
0
-HB
+HB
Supplemental Figure 11. HB treatments in tomato plants improve productivity under drought conditions in open field experiments. Total harvested fruits (A) and mean fruit weight (B) of tomato plants under limited water availability conditions (50%) and treated (+HB) or not (-HB) with 5mM HB. Time units are referred as follows: X DA Z, being X day (0-18); DA Days After; Z day of treatment (A-H). Points represent mean values. Different letters indicate significant differences at each time point (p < 0.05, two-way ANOVA with Tukey HSD). Number of harvested fruits (C) and total weight of harvested fruits (D) per categories were analyzed as follows: Class III-small size (25-50 mm diameter, 25-100 g weight); Class II-medium size (50-75 mm diameter,100-200 g weight); Class I-big size (75-100 mm diameter, 200-300 g weight); and Extra size (>100 mm diameter, >300 g weight).

## Slide 12
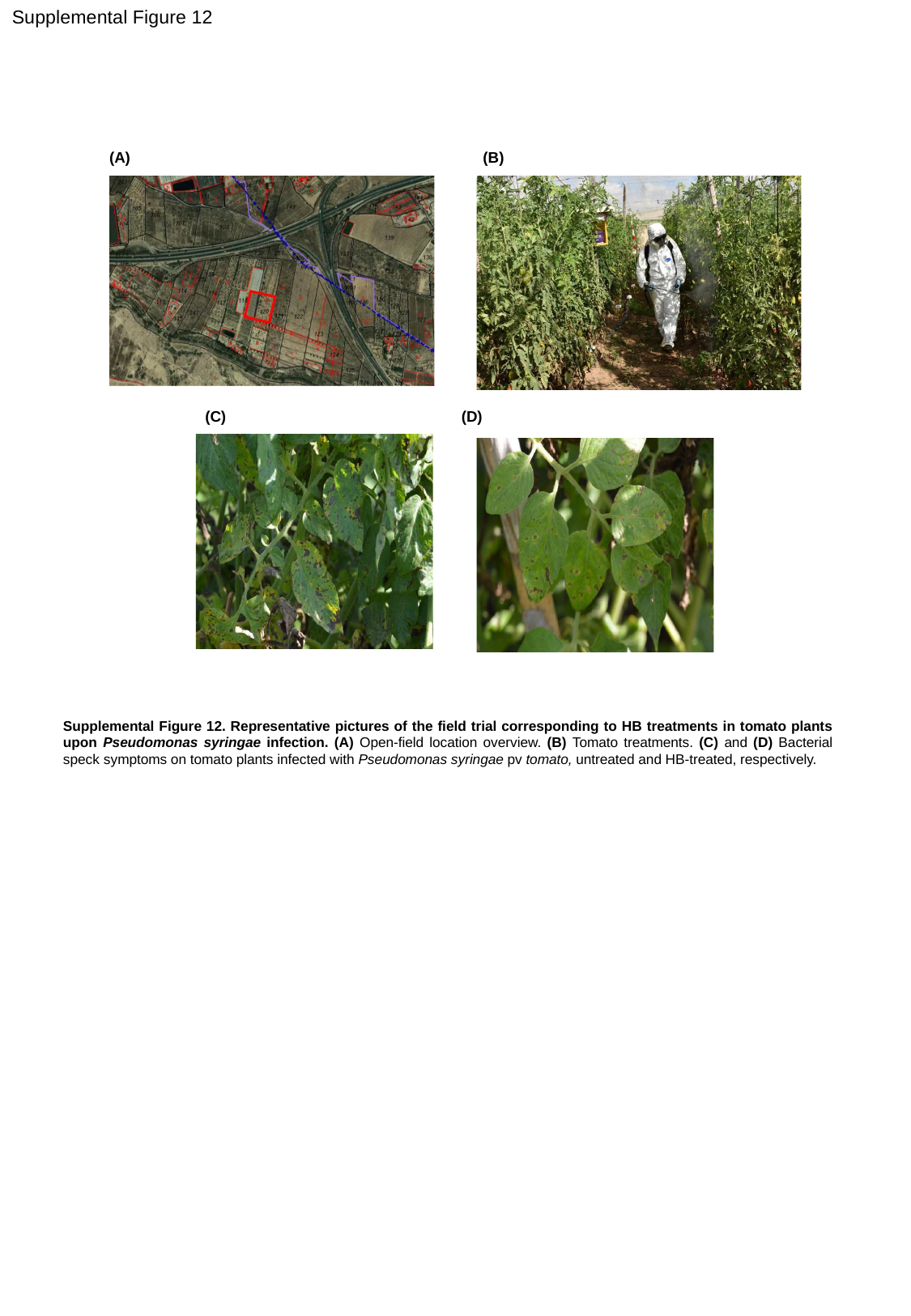

Supplemental Figure 12
(B)
(A)
(C)
(D)
Supplemental Figure 12. Representative pictures of the field trial corresponding to HB treatments in tomato plants upon Pseudomonas syringae infection. (A) Open-field location overview. (B) Tomato treatments. (C) and (D) Bacterial speck symptoms on tomato plants infected with Pseudomonas syringae pv tomato, untreated and HB-treated, respectively.

## Slide 13
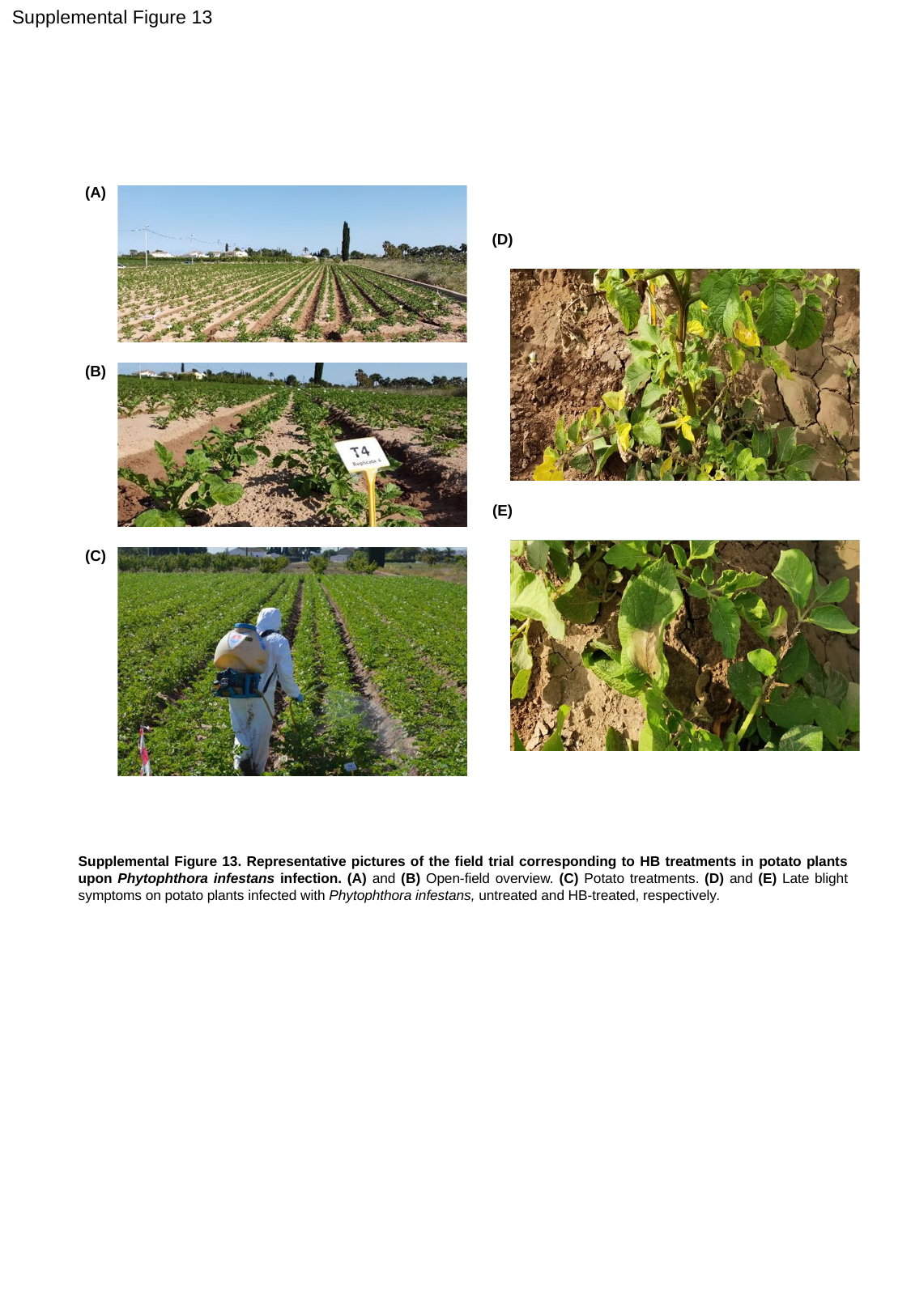

Supplemental Figure 13
(A)
(D)
(B)
(E)
(C)
Supplemental Figure 13. Representative pictures of the field trial corresponding to HB treatments in potato plants upon Phytophthora infestans infection. (A) and (B) Open-field overview. (C) Potato treatments. (D) and (E) Late blight symptoms on potato plants infected with Phytophthora infestans, untreated and HB-treated, respectively.

## Slide 14
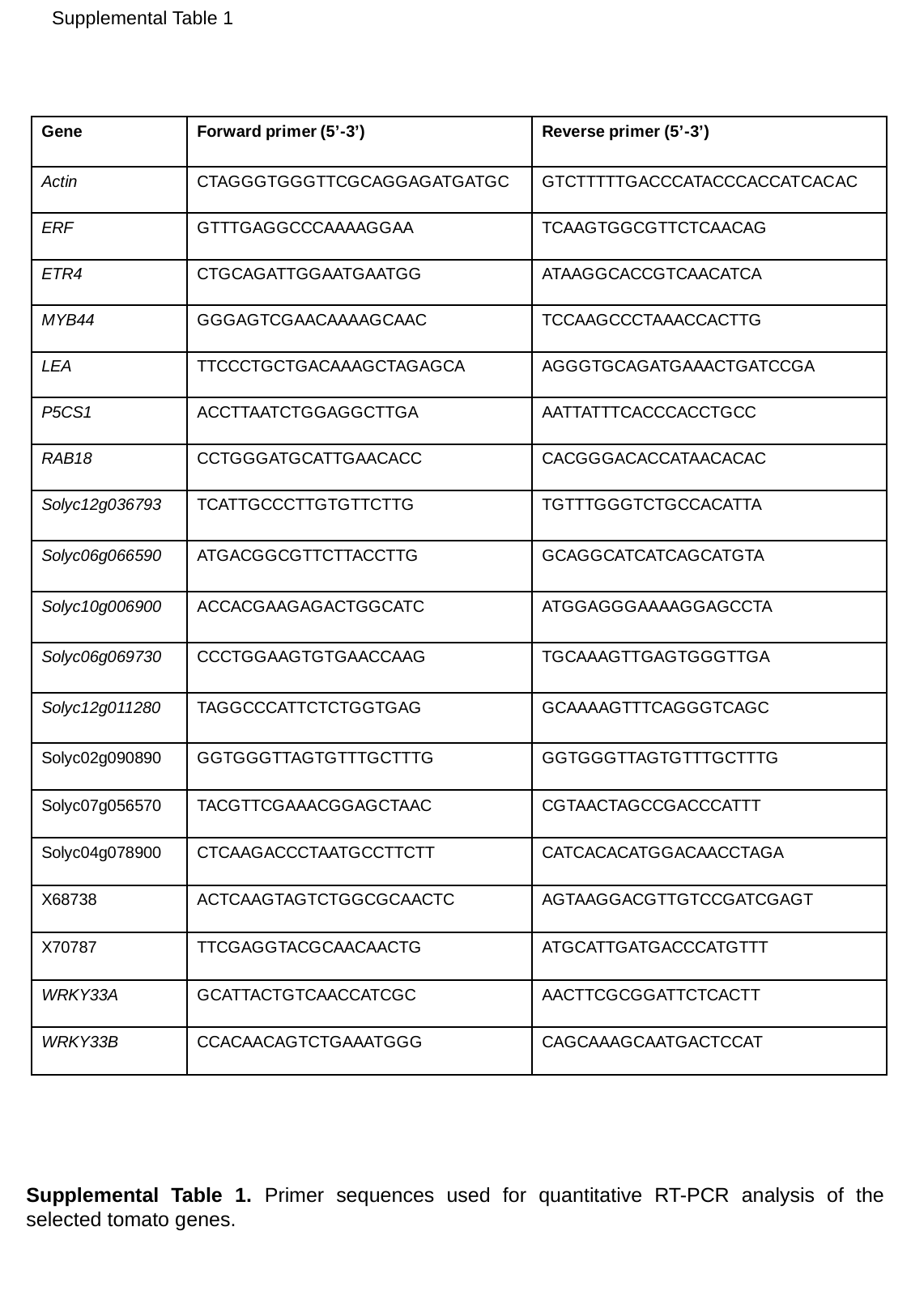

Supplemental Table 1
Supplemental Table 1. Primer sequences used for quantitative RT-PCR analysis of the selected tomato genes.
